# Supplementary material for: Polycystic Kidney Disease Ryanodine Receptor Domain (PKDRR) Proteins in Oomycetes
Source: Pathogens. 2020 Jul 16;9(7):577. doi: 10.3390/pathogens9070577 (PMC7399828; doi:10.3390/pathogens9070577)

**Supplemental Figure 1.** Potential mechanisms explaining the discrepancy between the apparent (101 kDa) and calculated (159 kDa) molecular weight of *P.infestans* PKDRR B protein. **A)** shows the intron-exon structure of the gene encoding PKDRR B. None of the potential translation products match the apparent molecular weight estimated by SDS-PAGE. **B)** Highlighted with a grey background is a putative intein of *P. infestans* PKDRR B. This is delimited by an N-terminal cysteine residue (C) and a consensus C-terminal HDS tripeptide. It contains a poorly conserved homing endonuclease domain and is of a suitable molecular weight to explain the discrepancy in the observed molecular weight of the PKDRR B protein (57 kDa). Also show are the peptide sequences used as immunogens for the generated of pAb XP1, XP2 and XP3 (the pAb XP1 peptide is located within the candidate intein).


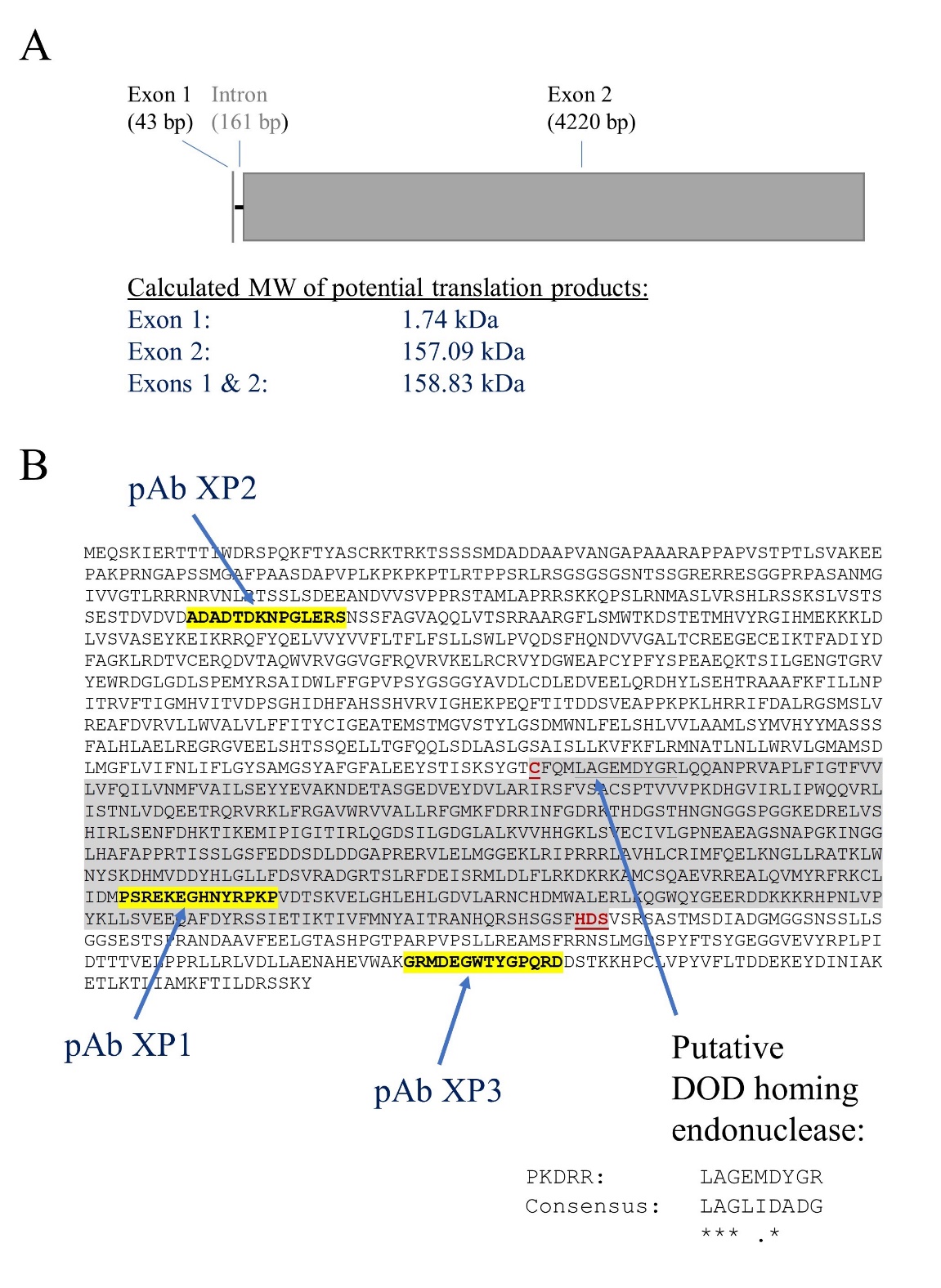

Supplement: Supplementary file 1 [file pathogens-09-00577-s001.zip › 845733 Figure S1 R2.docx]
