# Supplementary material for: Polycystic Kidney Disease Ryanodine Receptor Domain (PKDRR) Proteins in Oomycetes
Source: Pathogens. 2020 Jul 16;9(7):577. doi: 10.3390/pathogens9070577 (PMC7399828; doi:10.3390/pathogens9070577)
Supplement: Supplementary file 1 [file pathogens-09-00577-s001.zip › 845733 Figure S2 R3.docx]

Supplemental Figure 2. Manufacturer’s (GenScript) and BLASTP homology search data used to select immunogenic peptides for production of anti-PKDRR B Antisera*.* A) The position, sequence, length, antigenicity, surface probability, hydrophilicity, presence of a helix (coil), amphipathicity and ease of efficient synthesis are shown. The sequences of these synthetic peptides have an additional cysteine residue (C) at either the N- or C-terminus, to facilitate coupling to a carrier protein, which promotes immune response (indicated by “+1” in the “Length” column). B) Also shown are the results of BLASTP searches against *P. infestans* and *H. sapiens*. Each peptide (labelled “Query” on the alignments) is displayed aligned to its top two matches (“Sbjct”) in *P.infestans* and its best match in *H.sapiens*. Also shown are the calculated molecular weight of the subject protein, and its % identity and Expected probability (“Expect” or E-value) with the peptide. For short sequences, E-values of greater than 1e^-4^ are considered insignificant.

**A)**

| **No** | **Start** | **Antigenic Determinant** | **Length** | **Antigenicity/**  **Surface/**  **Hydrophilicity** | **Coil** | **Amphipathic** | **Synthesis** |
| --- | --- | --- | --- | --- | --- | --- | --- |
| **XP2** | 1359 | CGRMDEGWTYGPQRD | 14 + 1 | 3.05/1.00/1.04 | Y | N | N |
| **XP3** | 220 | ADADTDKNPGLERSC | 14 + 1 | 3.36/0.93/1.06 | Y | Y | N |
| **XP1** | 1124 | PSREKEGHNYRPKPC | 14 + 1 | 2.63/1.00/1.22 | Y | Y | N |

B)

**---------------------------------------------------------------------------**

**PEPTIDE XP1**

**---------------------------------------------------------------------------**

**Best match:** RyR domain [*Phytophthora infestans*] = ***P.infestans* PKDRR B**

Sequence ID: [KAF4041108.1](https://www.ncbi.nlm.nih.gov/protein/KAF4041108.1?report=genbank&log$=protalign&blast_rank=1&RID=ETRFD8SW014) Length: 1420 (Calculated MW: 160 kDa)

| **Score** | **Expect** | **Identities** | **Positives** | **Gaps** |
| --- | --- | --- | --- | --- |
| 49.4 bits(109) | **2e-09** | 14/14(**100%**) | 14/14(100%) | 0/14(0%) |

Query 1 PSREKEGHNYRPKP 14

PSREKEGHNYRPKP

Sbjct 1124 PSREKEGHNYRPKP 1137

**Second best match**: 6-pyruvoyl tetrahydropterin synthase domain-containing protein [*Phytophthora infestans*]

Sequence ID: [KAF4039674.1](https://www.ncbi.nlm.nih.gov/protein/KAF4039674.1?report=genbank&log$=protalign&blast_rank=4&RID=ETRFD8SW014) Length: 236 (Calculated MW: 26 kDa)

| **Score** | **Expect** | **Identities** | **Positives** | **Gaps** |
| --- | --- | --- | --- | --- |
| 24.0 bits(49) | 2.4 | 8/10(80%) | 8/10(80%) | 1/10(10%) |

Query 3 REK-EGHNYR 11

REK GHNYR

Sbjct 98 REKLHGHNYR 107

**Best match in *Homo sapiens*:** immunoglobulin heavy chain junction region [*Homo sapiens*]

Sequence ID: [MCD34086.1](https://www.ncbi.nlm.nih.gov/protein/MCD34086.1?report=genbank&log$=protalign&blast_rank=1&RID=ETR5REGN014) Length: 18 (Calculated MW (full protein): 75 kDa)

| **Score** | **Expect** | **Identities** | **Positives** | **Gaps** |
| --- | --- | --- | --- | --- |
| 25.2 bits(52) | 3.8 | 7/8(88%) | 7/8(87%) | 0/8(0%) |

Query 3 REKEGHNY 10

R KEGHNY

Sbjct 3 RGKEGHNY 10

**---------------------------------------------------------------------------**

**PEPTIDE XP2**

**---------------------------------------------------------------------------**

**Best match:**Ryanodine-inositol 1,4,5-triphosphate receptor Ca2 channel (RIR-CaC) family protein [*Phytophthora infestans* T30-4]= ***P.infestans* PKDRR B**

Sequence ID: [XP_002908895.1](https://www.ncbi.nlm.nih.gov/protein/XP_002908895.1?report=genbank&log$=protalign&blast_rank=2&RID=ETMCG7HF016) Length: 1420 (Calculated MW: 160 kDa)

Number of Matches: 2

| **Score** | **Expect** | **Identities** | **Positives** | **Gaps** |
| --- | --- | --- | --- | --- |
| 52.0 bits(115) | **3e-10** | 14/14(**100%**) | 14/14(100%) | 0/14(0%) |

Query 1 GRMDEGWTYGPQRD 14

GRMDEGWTYGPQRD

Sbjct 1359 GRMDEGWTYGPQRD 1372

| **Score** | **Expect** | **Identities** | **Positives** | **Gaps** |
| --- | --- | --- | --- | --- |
| 23.5 bits(48) | 3.4 | 7/13(54%) | 10/13(76%) | 0/13(0%) |

Query 2 RMDEGWTYGPQRD 14

R+ +GW YG +RD

Sbjct 1167 RLKQGWQYGEERD 1179

**Second best match:** RyR domain [*Phytophthora infestans*]

= ***P.infestans* PKDRR A**

Sequence ID: [KAF4031596.1](https://www.ncbi.nlm.nih.gov/protein/KAF4031596.1?report=genbank&log$=protalign&blast_rank=1&RID=ETMCG7HF016) Length: 1335 (Calculated MW: 152 kDa)

Number of Matches: 5

| **Score** | **Expect** | **Identities** | **Positives** | **Gaps** |
| --- | --- | --- | --- | --- |
| 29.5 bits(62) | 0.025 | 7/13(54%) | 11/13(84%) | 0/13(0%) |

Query 2 RMDEGWTYGPQRD 14

RM++GW +GP R+

Sbjct 1146 RMEQGWKFGPRRN 1158

| **Score** | **Expect** | **Identities** | **Positives** | **Gaps** |
| --- | --- | --- | --- | --- |
| 24.0 bits(49) | 2.4 | 8/14(57%) | 10/14(71%) | 0/14(0%) |

Query 1 GRMDEGWTYGPQRD 14

GR D+GW G +RD

Sbjct 1031 GRIDQGWRWGTERD 1044

**Best match in *H.sapiens*:** skeletal muscle ryanodine receptor [*Homo sapiens*]

Sequence ID: [AAC51191.1](https://www.ncbi.nlm.nih.gov/protein/AAC51191.1?report=genbank&log$=protalign&blast_rank=2&RID=ETP7Z8ZT014) Length: 5038 (Calculated MW: 565 kDa)

| **Score** | **Expect** | **Identities** | **Positives** | **Gaps** |
| --- | --- | --- | --- | --- |
| 32.5 bits(69) | 0.016 | 9/13(69%) | 11/13(84%) | 0/13(0%) |

Query 2 RMDEGWTYGPQRD 14

R ++GWTYGP RD

Sbjct 885 RIEQGWTYGPVRD 897

---------------------------------------------------------------------------

**PEPTIDE XP3**

---------------------------------------------------------------------------

**Best match:** Ryanodine-inositol 1,4,5-triphosphate receptor Ca2 channel (RIR-CaC) family protein [*Phytophthora infestans* T30-4] = ***P.infestans* PKDRR B**

Sequence ID: [XP_002908895.1](https://www.ncbi.nlm.nih.gov/protein/XP_002908895.1?report=genbank&log$=protalign&blast_rank=2&RID=ETE7TW3E016) Length: 1420 (Calculated MW: 160 kDa)

| **Score** | **Expect** | **Identities** | **Positives** | **Gaps** |
| --- | --- | --- | --- | --- |
| 46.0 bits(101) | **3e-08** | 14/14(**100%**) | 14/14(100%) | 0/14(0%) |
|  |  |  |  |  |

Query 1 ADADTDKNPGLERS 14

ADADTDKNPGLERS

Sbjct 220 ADADTDKNPGLERS 233

**Next best match:** Formin Homology 2 Domain [*Phytophthora infestans*]

Sequence ID: [KAF4042223.1](https://www.ncbi.nlm.nih.gov/protein/KAF4042223.1?report=genbank&log$=protalign&blast_rank=3&RID=ETE7TW3E016) Length: 1742 (Calculated MW: 186 kDa)

| **Score** | **Expect** | **Identities** | **Positives** | **Gaps** |
| --- | --- | --- | --- | --- |
| 23.5 bits(48) | 3.4 | 9/14(64%) | 11/14(78%) | 2/14(14%) |
|  |  |  |  |  |

Query 2 DA-DTDKNPGLERS 14

DA +T+ N GLERS

Sbjct 325 DALNTNRN-GLERS 337

**Best match in *H.sapiens*:** breast cancer 1, early onset, isoform CRA_l [Homo sapiens]

Sequence ID: [EAW60939.1](https://www.ncbi.nlm.nih.gov/protein/EAW60939.1?report=genbank&log$=protalign&blast_rank=3&RID=ETET7WE6016) Length: 680 (Calculated MW: 76 kDa)

| **Score** | **Expect** | **Identities** | **Positives** | **Gaps** |
| --- | --- | --- | --- | --- |
| 24.8 bits(51) | 8.5 | 10/16(63%) | 10/16(62%) | 2/16(12%) |
|  |  |  |  |  |

Query 1 ADADTDKN--PGLERS 14

AD T KN PG ERS

Sbjct 298 ADSSTSKNKEPGVERS 313
