# Supplementary figures and images for: Polycystic Kidney Disease Ryanodine Receptor Domain (PKDRR) Proteins in Oomycetes
Source: Pathogens. 2020 Jul 16;9(7):577. doi: 10.3390/pathogens9070577 (PMC7399828; doi:10.3390/pathogens9070577)

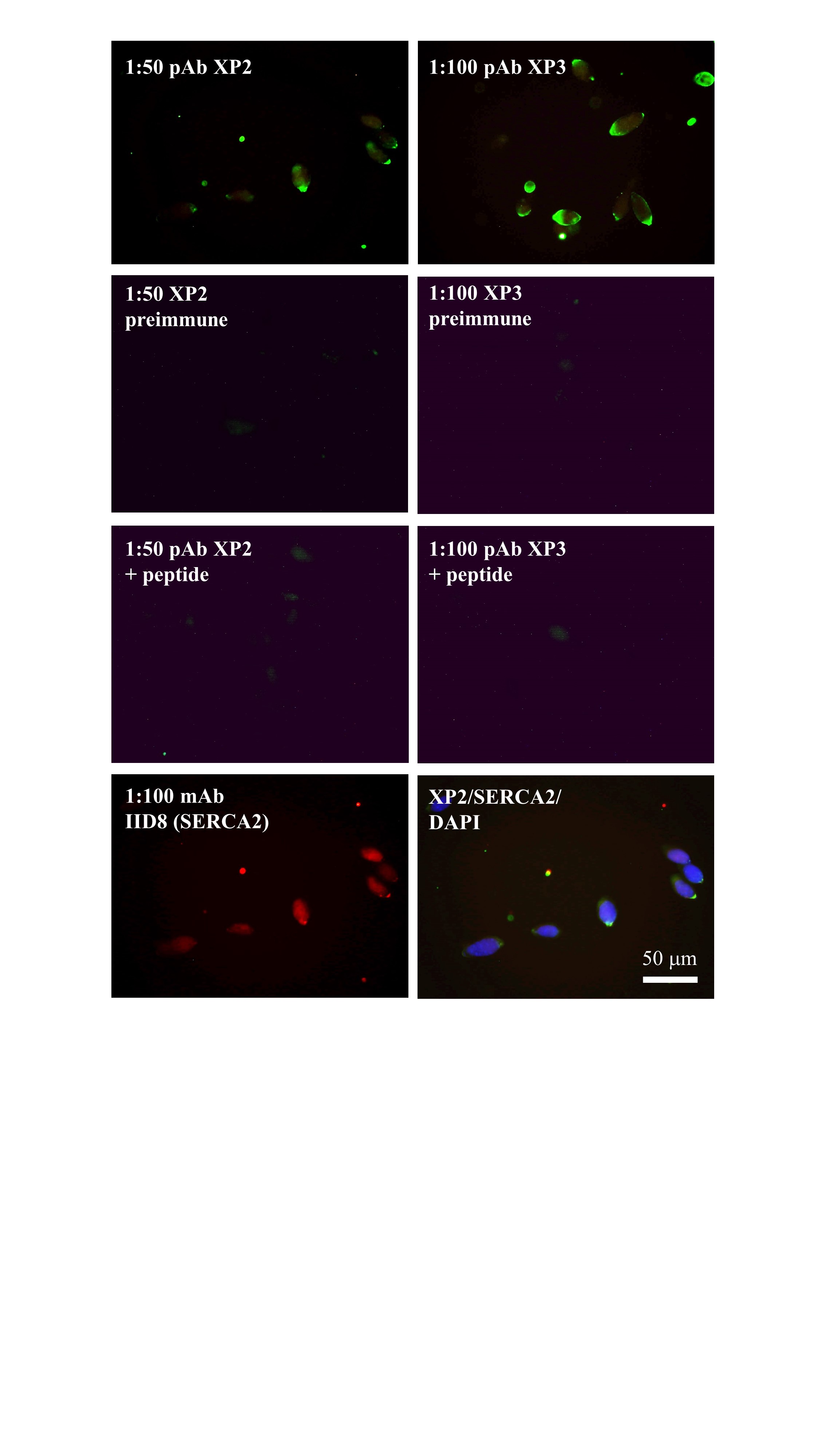

Supplement: Supplementary file 1 [file pathogens-09-00577-s001.zip › 845733 Figure S3 R2.jpg]
